# Hepatobiliary Cystadenoma Revealed by a Jaundice: A Case Report

**DOI:** 10.1155/2011/895605

**Published:** 2011-09-18

**Authors:** Taoufiq Harmouch, Marie-pierre Vullierme, Alain Sauvanet, Valérie Paradis, Affaf Amarti

**Affiliations:** ^1^Department of Pathology, University Hospital Hassan II, Fez 30000, Morocco; ^2^Department of Radiology, University Hospital Baujon, 92110 Clichy, France; ^3^Department of Surgery, University Hospital Baujon, 92110 Clichy, France; ^4^Department of Pathology, University Hospital Baujon, 92110 Clichy, France

## Abstract

*Introduction*. Hepatobiliary cystadenomas are rare benign cystic tumors and have a potential for recurrence and malignant transformation. The diagnosis may be very difficult because of absence of typical imaging feature in some cases. *Case Presentation*. In this paper, the authors discuss a 57-year-old woman who presented a jaundice related to hepatobiliary cystadenoma. Biological and radiological examinations have led to surgery, and the diagnosis is made after a histological examination of surgical specimens. *Conclusion*. This observation illustrates a hepatobiliary cystadenoma revealed by jaundice. Histology examination contributed to diagnosis. The authors discussed the mechanisms of biliary obstruction and differential diagnoses through a review of the literature.

## 1. Introduction

Hepatobiliary cystadenomas (HBCs) are rare benign cystic tumors [1], occurring in most of cases in women. The majority of HBCs are localized in liver parenchyma where the right side localization prevails in reports. They are usually incidentally findings, discovered by imaging diagnostic techniques [2]. In a minority of cases, clinical and biological manifestations may reveal these lesions. In that context, several differential diagnosis are possible, including hydatid cyst and liver metastasis [2, 3]. The accurate diagnoses of HBC is of crucial importance since complete excision of the lesion is mandatory to avoid both recurrence and malignant degeneration [2]. We describe here the clinical, radiological, and pathological features of one case of HBC characterized by the presence of a benign tumoral bud protruded into principal bile ducts. 

## 2. Case Presentation

**Figure 1:**
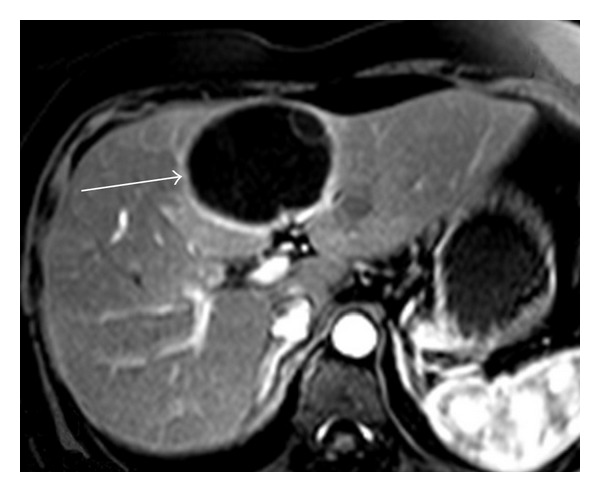
Axial T1 after gadolinium. Cyst in the segment IV of the liver with a thick enhanced wall (arrow).

**Figure 2:**
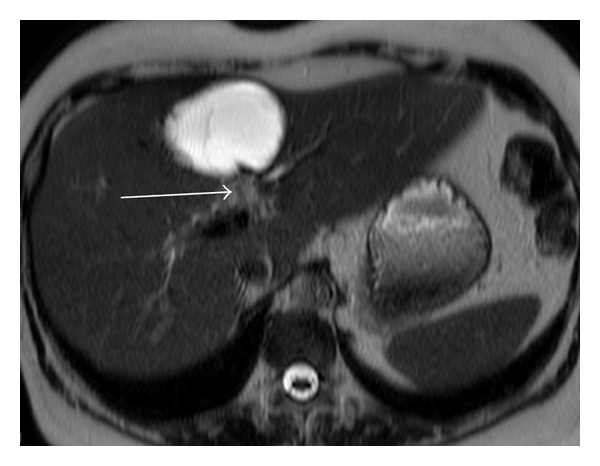
Axial T2 hyperintense cyst with a one-centimeter tissular lesion behind the caudal portion of the cyst (arrow).

**Figure 3:**
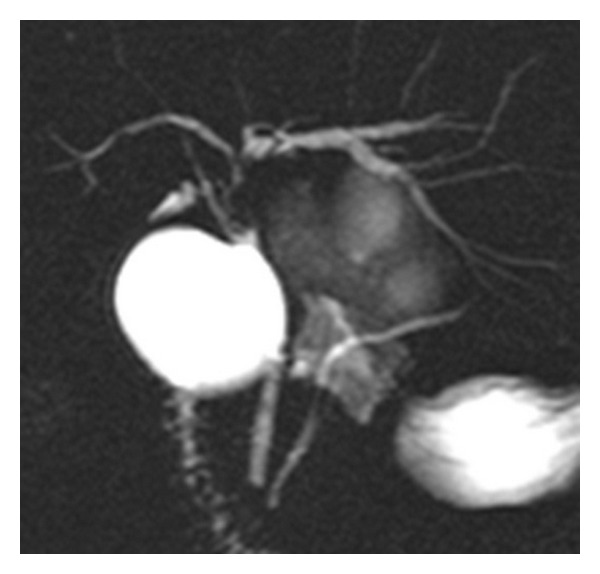
Thick slice cholangioMR. Mural nodule protruding in the left hepatic bile duct with upstream enlargement of this duct.

A 57-year-old woman was admitted to our hospital for investigation of epigastric and right hypochondriac pain and jaundice. Laboratory test results revealed liver dysfunction (cytolysis: alanine aminotransferase (ALAT) 922 IU/L; cholestasis: glutamyl transpeptidase (GGT) 1705 IU/L; alkaline phosphatase (PAL) 177 IU/L). The hydatid serology was negative. Liver MRI with cholangioMR was performed (Figures 1, 2, and 3). Axial T1 postgadolinium slices showed a cyst in the segment IV of the liver with a thick enhanced wall, axial T2 showed a one-centimeter tissular lesion behind the caudal portion of the cyst, and thick slice cholangioMR showed a mural nodule protruding in the left bile duct.

**Figure 4:**
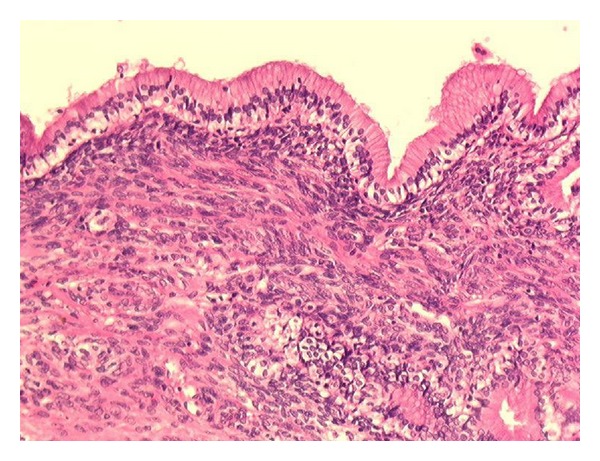
Mucinous cystadenoma with mesenchymal stroma: mucus-secreting epithelium resting on a stroma rich in mesenchymal cells.

In the draining cyst, fluid analysis found elevated carbohydrate antigen CA19.9 (higher than 70 000 U/mL), and carcinoembryonic antigen (CEA: 5.8 ng/mL) without any scolex or other pathogenic agents. The proposed diagnosis was a cystadenoma probably communicating with biliary ducts. Intraoperative endoscopic retrograde cholangiography revealed a bud into the left extrahepatic bile duct. Surgery consists on a left hepatectomy associated with cholecystectomy and intrabiliary tumoral bud resection. Both upper biliary confluence and common bile duct were free of tumor and note resected. Macroscopically, two cystic masses of 4.5 × 5 cm and 1.5 × 1.5 cm large were found in segment IV of the liver. The inner wall lining was smooth, without any infiltrative pattern. The cystic cavities were filled with clear mucinous fluid. Histopathological analysis confirmed the diagnosis of typical HBC harboring mesenchymal stroma (Figure 4), without any features of malignancy. Histopathological analysis of intrabiliary tumoral bud showed the same morphological pattern, consisting of a bud lined by cuboïdal epithelium lining and mesenchymal stroma.

## 3. Discussion

HBCs represent approximately 5% of intrahepatic cystic lesions [4]. Among them, HBC displaying mesenchymal stroma are well-defined entity developed almost exclusively in women. We report here a case of benign HBCs with mesenchymal stroma which grew into biliary system, leading thus to jaundice. Such clinical presentation is very rare and radiologists and surgeons must be aware of it. Clinically, these tumors have the same presentation as other hepatobiliary masses. The differential diagnosis includes surinfected hepatic cysts, liver pyogenic abscesses, hydatid cysts, cystic degeneration of any liver tumour, Caroli's disease, and posttraumatic and hemorrhagic cysts. The radiological studies may reveal the difference between biliary cystadenoma and other simple cystic lesions by the presence of septations and irregularly thickened cyst walls with or without calcifications. The internal septa and wall are enhanced with intravenous contrast. A solid component in the cyst, intracystic papillary projections, or calcifications may suggest malignancy. Commonly used imaging modalities include ultrasounds, CT scan, and MRI. Macroscopically, HBCs form multilocular cystic tumors filled with mucinous fluid and surrounded by irregularly thick walls [4]. Histologically, HBC with mesenchymal stroma are differentiated from other cystadenomas by the presence of a primitive mesenchymal stroma [5]. On histogenesis ground, HBC are supposed to develop either from foci of primitive hepatobiliary cells, or from normal intrahepatic ducts which probably develop neoplastic changes after stimulation by some still unknown causes [6]. Jaundice can reveal HBC and can be due to distinct mechanisms. HBC with invasive malignant component can directly invade the upper confluence or can be associated with metastasic lymph nodes of the hepatic pedicle compressing the common bile duct. Our case illustrates a more specific and rarer mechanism of jaundice: a communication of the HBC with the biliary tree. Such complication, previously reported in only few cases, could be due to direct tumor growth of the tumors into biliary duct system. This latter hypothesis is supported by anatomy of bile duct drainage [6]. As a matter of fact, the duct from segment IV often joins the left hepatic duct perpendicularly. This might be considered as a predisposing situation which could be followed by protrusion of HBC into the left hepatic duct [7]. In our case, segment IV localization of cystadenoma can explain a tumoral bud protruded into left principal bile ducts.

## 4. Conclusion

The presence of thick wall, endoluminal buds, and septations at imaging investigation is suggestive of the diagnosis of HBC. The diagnosis may be very difficult because of absence of typical imaging feature in some cases, or sometimes with unusual clinical presentation such as jaundice. The pathology confirmation is needed for optimal treatment.

##  Conflict Interests

The authors declare that they have no conflict of interests.
